# Prevalence and determinants of biochemical dysfunction of the liver in Atayal Aboriginal community of Taiwan: Is betel nut chewing a risk factor?

**DOI:** 10.1186/1471-230X-8-13

**Published:** 2008-04-27

**Authors:** Ching-Feng Lin, Tun-Jen Shiau, Ying-Chin Ko, Ping-Ho Chen, Jung-Der Wang

**Affiliations:** 1 Institute of Occupational Medicine and Industrial Hygiene, National Taiwan University, College of Public Health and Department of Internal Medicine, National Taiwan University Hospital, Taipei, Taiwan; 2 Department of Health, Executive Yuan, Taiwan; 3 Department of Public Health, Faculty of Medicine, College of Medicine, Kaohsiung Medical University, Kaohsiung, Taiwan; 4 Division of Environmental Health and Occupational Medicine, National Health Research Institutes, Taiwan; 5 School of Public Health, Taipei Medical University, Taiwan

## Abstract

**Background:**

We address the independent and interactive roles of habitual betel quid chewing and other known risk factors for biochemical dysfunction and cirrhosis of the liver.

**Methods:**

To determine the prevalence rates and risk factors associated with biochemical dysfunction of the liver, a total of 3,010 adult residents in an Atayal Aboriginal community were invited to participate in the study. Abdominal ultrasonography was used to diagnose liver cirrhosis.

**Results:**

There were 2,063 Atayal Aboriginal and 947 non-Aboriginal in this study. The result showed overall prevalence rates for hepatitis B surface antigen (HBsAg) and hepatitis C virus (HCV) were 21.2 % and 2.9 %, respectively. There were 16.5 %, 15.1 % and 22.4 % subjects with abnormal alanine aminotransferase (ALT), aspartate aminotransferase (AST), and gamma glutamyl transpeptidase (GGT), accordingly. Multiple logistic regression analysis showed that combined infections with HBV and HCV presented with the highest risks with OR (odds ratio) and 95% CI (confidence interval) of 4.2 (1.2–17.4) and 3.8 (1.0–14.1), respectively for elevation of ALT and AST; followed by alcohol (1.7 and 3.1), male gender (1.7 and 1.6), betel quid (1.5 and 1.3), smoking (1.4 and 1.8), and aboriginal (1.4 and 1.3). There is effect-measure modification between viral infection and betel quid chewing for increased severity of abnormal ALT elevation. Among 1,382 subjects consenting to abdominal ultrasonography, 41(3.0%) were found to have liver cirrhosis with the same factors associated with higher risks.

**Conclusion:**

In addition to infections with viral hepatitis B and/or C, we found Atayal Aboriginal, males, current smokers, drinkers and betel quid chewers were independently associated with biochemical dysfunction and probably cirrhosis of the liver. Further study is needed to corroborate the above hypothesis.

## Background

Health inequity and disparities have been demonstrated to exist between Aboriginal and non-Aboriginal in the USA, Australia, Taiwan and other countries [1]. In Australia, for almost all disease categories, rates for Aboriginal were higher than those for other Australians, which were mainly attributed to the hypothesis of social and economic inequality [2-4]. Such an inequality may result in an increase of not only infectious diseases but also almost every kind of diseases associated with diet, exercise patterns, apathy and health behavior, such as alcohol drinking, cigarette smoking, betel quid chewing and obesity [1].

The population of Taiwan is 23 million, consisting of Hokkien (73%), Hakka (12%), Mainland Chinese (13%), and Aboriginal (2%) ethnic groups [5]. There are twelve Aboriginal tribes in Taiwan and constitute approximately 460,000 Aboriginal of the Taiwanese (general) population. Although there have been long term mixed marriage with other ethnic groups, Taiwan Aboriginal still preserved their own languages, customs and social organizations. In hereditary characteristics, they are markedly different from the rest of Taiwanese population [6]. Their health status, as evaluated by life expectancy, mortality rates and the prevalence and incidence of various diseases such as accident, liver disease and other diseases, was worse than that of the general population in Taiwan [1]. A more recent investigation indicated that the discrepancy of life expectancy between Aboriginal and non-Aboriginal were slightly reduced, but the life expectancy of Aboriginal were on average 10 years less than that of the general population, with a standardized mortality ratio approaching 2-fold [7].

Taiwan is known for the high prevalence of chronic hepatitis B virus (HBV) infection, with a positive rate of 15–20 % for hepatitis B surface antigen (HBsAg) [8,9], which was also reported among Aboriginal people [8]. The prevalence of hepatitis C infection was around 2–4 % [10]. Aboriginal in Taiwan were also found to have a high prevalence rate of alcoholism, estimated at about 20–30 % [11,12], which may result in abnormal liver function or even chronic liver injury. Thus, abnormal liver function in Aboriginal people in Taiwan has usually been considered to be virus or alcohol related.

Betel quid chewing is a prevalent habit in 10–20% of the general population [13], and is also part of traditional Aboriginal culture usually taken with alcohol during festival or traditional ceremony in Taiwan [14]. The betel quid prepared in Atayal aboriginal community of Taiwan is quite different from those in other parts of the world. It usually consists of 2 halves of a fresh areca nut (fruit of the *Areca catechu *Linn.), sandwiched with a piece of the betel leaf, and white slaked lime paste, instead of the reddish inflorescence. Betel quid chewers generally swallow the saliva completely, thus bathing the epithelial lining of the upper digestive tract with the toxins released during chewing. Such a practice may increase the possibility of toxic effects of betel quid at target organs other than the oral cavity [15]. In 2004, the International Agency for Research on Cancer (IARC) declared chewing of betel quid, by itself, to be a Group 1 carcinogen and the areca nut to be, correspondingly, a Group 1 carcinogen [16]. Chewing betel quid independently contributes to the risk of oropharyngeal cancer [17-19], oral mucosal lesions [20], oral leukoplakia [21], oral submucous fibrosis (OSF) [21], liver cirrhosis [22,23], hepatocellular carcinoma (HCC) [24], diabetes mellitus [25] and adverse outcomes with use during pregnancy [26]. Endogenous formation of areca-nut-derived nitrosamines was found *in vivo *or *in vitro *studies, suggesting that the damage of liver could partly result from metabolite of the areca nut [27].

Chronic inflammation of the liver appears to be a risk factor for cirrhosis regardless of the underlying etiology [28-30]. Experimental study has indicated persistent hepatocellular damage after chronic feeding with betel quid [31,32]. However, the role of betel quid chewing in the development of liver injury and/or cirrhosis, and its interaction with other known risk factors, have not drawn much attention, although Lin et al and Tsai et al independently published case-control studies with cirrhosis and/or chronic liver diseases suspected to be associated with this habit [22,23]. In this study we address the independent and interactive roles of habitual betel quid chewing and other known risk factors for biochemical dysfunction and cirrhosis of the liver.

## Methods

### Design

There are 12 linguistically distinct Aboriginal tribes in Taiwan, which constitutes about 2 % of the total Taiwanese population. They mainly reside in the eastern plains and central mountainous area where is mainly above a height of 500 m. The ethnic status was classified into two groups, "Aboriginal" and "non-Aboriginal". Aboriginal were defined when at least one of the two parents was Atayal Aboriginal. Also, non-Aboriginal were defined when both parents were not Atayal Aboriginal. The non-Aboriginal in this study were mainly Hokkien and Hakka people living in the same community. In this study, we selected the Fushin district in Taoyuan county, which was in the catchment area of our investigations and locates in the northern part of Taiwan with a total of 4,665 residents who were above 20 years of age. Every citizen in the district was invited to participate in a comprehensive physical examination including a detailed questionnaire of health history. A total of 3,010 subjects (64.5%) responded, which included 2,063 Atayal Aboriginal and 947 non-Aboriginal.

This study was first approved by the medical research ethics committee of the hospitals of the Department of Health of Taiwan before commencement, of which the certification number is 900808-03. The informed consent was provided both orally and in writing, which was conducted under the assistance of local public health nurses who can speak Atayal language.

### Data collection and analysis

After obtaining an informed consent from every subject, 10 c.c. of blood was drawn from every respondent's ante-cubital vein. All blood samples were centrifuged and serum was frozen down to – 30°~-70°C until they were tested for hepatitis B & C markers and biochemical function of the liver, including alanine aminotransferase (ALT), aspartate aminotransferase (AST), and gamma-glutamyltranspeptidase (GGT).

Test for HBsAg and anti hepatitis C virus (HCV) was conducted by radioimmunoassy with reagents supplied by Abbott company (U.S.A.), and read by clinical Gamma Counter (L.K.B of U.S.A.) with both positive and negative controls. If the reading was on the borderline, the test was repeated 2~3 times. If it consistently showed positive, then the result was considered positive. Liver function tests were performed with reagents supplied by Hitachi 7400. All these serological tests were conducted in the central laboratory of the Taoyuan general Hospital, Department of Health. Every subject was also asked to fill out a questionnaire containing basic demographic information and previous medical history, ethnic group, alcohol drinking, smoking, betel quid chewing, blood transfusion and intravenous injection. Every case was physically examined by a doctor. The history of blood transfusion and intravenous injection was verified from the medical records of the local public health stations, which were the only resource providing health services to the people residing in mountainous area and the data were with a confirmed rate of 99%. Every subject was also invited to receive abdominal ultrasonography examination (Toshiba SAL-38B, Tokyo, Japan).

#### Dependent variables

Separate analyses were conducted for biochemical liver function (ALT, AST, GGT) and liver cirrhosis. The dichotomous variables, biochemical liver function, were coded as ALT (>=37; < 37)/AST (>=37; < 37)/GGT (>=61/<61). Data was separately analyzed for risk factors associated with biochemical liver function/liver cirrhosis. The only one hepatologist conducted all the real time ultrasonography and scored on 10 parameters [33], which ranged from 0, 0.5, 1(mild), 2(moderate), and 3(marked) for each parameter. The more specific parameters for cirrhosis included nodular liver surface, coarse echotexture, irregular narrowing of hepatic veins and a summed score of more than 4.0 indicated cirrhosis of liver with an accuracy of 95.6% [33].

#### Independent variables

Potential risk factors included ethnic group, gender, age, body mass index (BMI), cigarette smoking, alcohol consumption, betel quid use, HBsAg(+), and anti-HCV(+). Body mass index (BMI) was calculated (BMI = weight/height^2^, kg/m^2^). The smoking and drinking behaviors were coded as current smokers/drinkers (continual smoking/drinking least once a week, irrespective of quantity), never smokers/drinkers/(those who never smoked/drank), or former smokers/drinkers (those who quit smoking/drinking at least one year prior the survey). Betel quid chewing behavior, was coded as betel quid chewers (chewed at least once a week, irrespective of quantity) or never chewers (those who never chewed).

### Statistical analyses

All the information of medical history and laboratory results was collected and put into computer file and processed and analyzed by SAS version no. 6.02. A chi-square test was performed for each univariate analysis first to screen in potentially significant variables. Then, we calculated odds ratio (OR) with 95% CI of abnormal biochemical function of the liver through multivariate modeling after controlling major risk factors, including age, gender, BMI, ethnic group, alcohol drinking, cigarette smoking, betel quid chewing and hepatitis B & C status. A backward stepwise procedure was undertaken with a selection of p-value less than 0.001 to be kept in the final model.

## Results

Originally we invited 4,665 residents who were above 20 years of age to take part in the study. Some residents did not participate in the study, because they have already taken jobs in urban area and moved out of the village. In total, we successfully collected blood samples and personal information from 3,010 subjects in this Atayal Aboriginal community of Taiwan. Because our local public health nurses went on door-to-door visits to every household in the community, almost all people who still resided in the community were screened. 48 % of them were males. Table 1 depicted the distribution of risk factors associated with abnormal liver function by using a chi square test in the univariate analysis. The average age was 49.8 ± 10.2 years old, with about more than one third of them in the age range of 41~60 years old (36.9%), as summarized on Table 1. Among these participants, there were 2,063 (68.5%) Aboriginal, in which about one fifth showed an abnormal ALT, 18.2% showed an abnormal AST and 28.6% an abnormal GGT. Aboriginal consistently showed higher proportions of smoking, alcohol drinking, betel quid chewing, overweight, and abnormal transminases than did non-Aboriginal. In addition, cigarette smokers, alcohol drinkers, HBsAg carriers, people with habitual betel quid chewing, and people who were infected by HCV were found to have significantly higher proportions of abnormal liver transaminases (all *p *value < .001). The overall prevalence rates of HBsAg and anti-HCV were 21.2 % and 2.9%, respectively.

**Table 1:** Frequency of demographic factors and risk factors stratified by ethnicity, and abnormal elevations of biochemical functions (ALT, AST, and GGT) in an Atayal aboriginal community of Taiwan

	Ethnic groups				
				
	Aboriginal	Nonaboriginal	ALT>=37(%)	AST>=37(%)	GGT>=61(%)
No. of subjects	2063	947	496(16.5) *	454(15.1) *	675(22.4) *

Aboriginal			429(20.8) *	377(18.2) *	591(28.6) *
Age(year)					
≤ 20	3	1	1(25.0)	0(0)	1(25.0)
21–40	732*	262	191*(19.2)	194*(19.5)	240(24.1)
41–60	743	370	211(18.9)	190(17.0)	317*(28.5)
≥ 61	585	314	93(10.3)	70(7.8)	116(12.9)
Gender					
Male	992	460	357(24.5) *	321(22.1) *	508(34.9) *
Female	1071	487	139(8.9)	133(8.5)	167(10.7)
Cigarette smoking	930*	252	248(21.0) *	339(28.7) *	460(38.9) *
Alcohol drinking	1226*	267	295(19.8) *	410(27.5) *	577(38.6) *
Betel quid chewing	995*	122	399(35.7)*	375(33.6)*	290(26.0)
HBsAg(+)	441	197	153(24.0) *	147(23.1) *	154(24.1) *
Anti-HCV(+)	61	25	31(36.1) *	33(38.3) *	31(36.1) *
BMI>25	840*	277	384(34.4) *	341(30.5)	392(35.1)*

*p < 0.001 for crude chi-square test of association between individual risk factors and aboriginal ethnicity

Table 2 summarizes the results of multiple logistic regression modeling by using a backward stepwise procedure to choose the major risk factors (*p *value < 0.001) of abnormal biochemical liver function. After adjustment for gender, BMI, viral hepatitis B and C infections, smoking, alcohol drinking and betel quid chewing, we found that Aboriginal were more likely to develop abnormal elevation of liver transaminases than non-Aboriginal, with an OR of ALT 1.4 (95 % C.I., 1.2–1.7), AST 1.3 (95 % C.I., 1.1–2.2), and GGT 1.5 (95 % C.I., 1.2–2.9). We also found that betel quid chewing was independently associated with abnormal elevation of liver transaminases, with an OR of ALT 1.5 (95 % C.I., 1.1–1.8) and AST 1.3 (95 % C.I., 1.1–1.7). Current alcohol drinking increased likelihood of developing abnormal elevation of liver transaminases after controlling other risk factors, with 4.7 times of risk for GGT. Similarly, current smoking, male gender, and viral hepatitis B or C infection also showed a significantly increased likelihood of abnormal elevation of ALT, AST, GGT after controlling other risk factors as summarized on Table 2. Increased BMI (> 25) was also associated with increased risks for abnormality in ALT and GGT. There are linear trends for increased severity levels of ALT elevation for both hepatitis virus infection and betel quid chewing, and there was about 4.7 % (140/3010) of the study population with more than 5 times of elevation of ALT. After stratification by different levels of elevated ALT, the multiple logistic regression analysis also showed a synergistic effect between viral infection and betel quid chewing, as summarized in Table 3.

**Table 2:** Frequency, odds ratio(OR) and 95%CI (confidence interval) for different risk factors of abnormal biochemical liver function estimated from multiple regression logistic model after adjustment for age, gender, and education level

Variables	ALT		AST		GGT	
	No. of Cases		No. of Cases		No. of Cases	

	>=37/<37 OR (95% CI)		>=37/<37 OR(95% CI)		>=61/<61 OR(95% CI)	
Ethnic group						
Non-Aboriginal	68/868	1.0	66/870	1.0	84/852	1.0
Aboriginal	340/1724	1.4(1.2–1.7)	429/1635	1.3(1.1–2.2)	590/1472	1.5(1.2–2.9)
Alcohol drinking						
Never	113/1402	1.0	85/1430	1.0	97/1417	1.0
Former	16/127	1.1(0.6–2.0)	14/129	1.0(0.5–1.9)	16/129	1.0(0.5–1.9)
Current	279/1068	1.7(1.2–2.2)	396/951	3.1(2.4–4.3)	561/951	4.7(3.3–6.0)
Cigarette smoking						
Never	160/1666	1.0	156/1670	1.0	214/1612	1.0
Former	19/76	1.4(0.8–2.2)	22/73	1.7(1.2–3.1)	27/68	1.1(0.6–16)
Current	229/1068	1.4(1.1–1.8)	317/766	1.8(1.3–2.2)	433/649	1.6(1.2–2.1)
Betel quid chewing						
No	117/1584	1.0	129/1613	1.0	154/1857	1.0
Yes	282/1117	1.5(1.1–1.8)	246/983	1.3(1.1–1.7)	136/527	0.7(0.5–1.1)
Gender						
Female	117/1439	1.0	139/1417	1.0	167/1387	1.0
Male	291/1158	1.7(1.5–2.3)	356/1093	1.6(1.2–2.0)	507/942	2.0(1.9–2.9)
BMI						
<=25	214/1659	1.0	326/1547	1.0	374/1499	1.0
>25	193/929	1.9(1.5–2.4)	167/955	1.0(0.7–1.1)	295/825	1.9(1.5–2.3)
HBV/HCV						
-/-	232/971	1.0	299/1904	1.0	470/1731	1.0
+/-	132/496	1.8(1.4–2.3)	148/480	1.7(1.3–2.2)	148/480	0.8(0.6–1.0)
-/+	27/49	3.7(3.2–9.2)	26/50	3.1(2.1–6.2)	25/51	2.0(1.3–4.4)
+/+	5/5	4.2(1.2–17.4)	5/5	3.8(1.0–14.1)	6/4	2.3(0.7–11.0)

ALT:Alanine aminotrnsferase; AST:Alanine aminotrnsferase; GGT:γ-glutamyl transpeptidase; +:positive; -:negative; OR: odds ratio

**Table 3:** Frequencies and OR (odds ratio) and 95%CI (confidence interval) for betel quid chewing and infection with HBV/or HCV stratified by different severity levels of abnormal alanine transaminase (ALT) estimated from multiple logistic regression model after adjustments for alcohol and cigarette consumptions, age, ethnicity, BMI (body mass index) and gender

Levels of ALT elevation		≤37 [1 ×]	OR (95%CI)	>37–≤111 [1–3 ×]	OR (95%CI)	>111–≤185 [3–5 ×]	OR (95%CI)	>185 [>5 ×]	OR (95%CI)
Betel Quid Chewing	HBV/or HCV	No. of subjects		No. of subjects		No. of subjects		No. of subjects	
No	-	1005	1.0	135	0.9 (0.6–1.2)	40	0.5 (0.3–0.8)	23	0.3 (0.1–0.8)
	+	318	1.3 (1.2–1.5)	18	1.6 (1.2–1.9)	21	1.8 (1.3–2.3)	24	2.3 (2.1–2.7)
Yes	-	935	1.1 (0.8–1.5)	31	1.3 (1.1–1.3)	23	1.5 (1.2–1.9)	28	1.7 (1.4–2.1)
	+	256	1.5 (1.2–1.8)	36	2.3 (1.9–2.6)	52	3.1 (2.5–3.6)	65	4.2 (3.5–4.7)

HBV: HBsAg(+)

HCV: hepatitis C virus infection

+: positive

-: negative

OR(95%): odds ratio(95%CI)

1 ×: upper normal limit

1–3 ×: one to three times of upper normal limit

5 ×: five times of upper normal limit

Only 1382 subjects came for abdominal ultrasound examination, which detected 41 cases (3.0 %) with cirrhosis of liver. Among them, 4 cases of liver cancer were subsequently diagnosed and verified with pathology. All 4 cases were male Aboriginal with positive HBsAg and a habit of alcohol drinking, except one quitted about 10 years ago. The average age of subjects with cirrhosis of liver was 46 ± 14 years. Approximately 96 % of liver cirrhosis subjects had a history of alcohol drinking (12.5 % of them have already quitted) and 45 % of them showed positive HBsAg. After controlling for other risk factors, we found that Aboriginal were associated with liver cirrhosis, with an adjusted OR of 1.8 (95 % C.I., 1.4–3.2), as shown in Table 4. Similarly, former and current alcohol drinking increased risks of developing cirrhosis of liver with 1.6 and 2.1 times, respectively. Betel quid chewing was independently associated with cirrhosis of liver, with an adjusted OR of 1.7 (95 % C.I., 1.2–2.3).

**Table 4:** Adjusted odds ratio(OR) and 95%CI (confidence interval) from multiple logistic regression for risk factors of cirrhosis of liver diagnosed by ultrasonography

Variables	No. of liver cirrhosis/total	OR (95%CI)
No. of Cases	41/1382 (3.0%)	
Ethnic group		
Non-Aboriginal	7/398 (1.8%)	1.0
Aboriginal	34/984 (3.5%)	1.8(1.4–3.6)
Alcohol drinking		
Never	2/681(0.3%)	1.0
Former	6/101(5.9%)	1.6(1.3–4.6)
Current	33/600(5.5%)	2.1(1.5–3.5)
Cigarette smoking		
Never	9/892(1.0%)	1.0
Former	2/49(4.1%)	1.3(0.6–10.7)
Current	30/441(6.8%)	1.8(1.1–2.7)
Betel quid chewing		
No	19/679(3.5%)	1.0
Yes	22/703(2.2%)	1.7(1.2–2.3)
Gender		
Female	5/732(0.7%)	1.0
Male	36/650(5.5%)	1.5(1.2–3.9)
BMI		
<=25	26/852(3.1%)	1.0
>25	15/530(2.8%)	0.9(0.6–1.7)
HBV/HCV		
-/-	21/1020(2.1%)	1.0
+/-	18/300(6.0%)	1.9(0.4–12.8)
-/+	1/32(3.1%)	2.2(1.4–4.7)
+/+	1/3(33.3%)	5.4(0.2–7.6)

+:positive; -:negative OR: odds ratio

## Discussion

Although the frequencies of abnormal ALT, AST, and GGT among Aboriginal were found to be all above 18% and significantly higher than in non-Aboriginal (all p's < 0.001), such associations might not be necessarily causal, especially using a cross-sectional design, requires careful evaluation. In this study, most people in this Aboriginal community were employed by industry related to tourism and sightseeing. It also attracted a lot of non-Aboriginal people to work and reside in the community and provided us an excellent opportunity to compare the findings in these ethnic groups. In fact, the proportions of betel quid chewers among Aboriginals (48.2%) and non-Aboriginals (12.8%) estimated from this study were similar to those reported before [12,16], indicating a relatively representative sample from both ethnic groups. Since the coverage proportions of National Health Insurance for non-Aboriginal and Aboriginal people of this community were about 97% and 93 %, respectively, and both of them were waived from partial co-payment, the likelihood of selection bias seems very low.

After controlling all known risk factors for liver diseases (including viral infections, smoking, alcohol drinking, overweight, gender and ethnicity), there was still 1.3 – 1.5 times of increased odds ratio in Aboriginal. Thus, the association deserves our further consideration. There were following possible random errors on measurements of determinants: The history of ethnic group was generally very accurate because only Aboriginals were entitled to a complete waiver for partial co-payment if treated by a doctor. The BMI of every subject was calculated by a nurse immediately after physical measurements of body weight and height. Thus, only the history of alcohol drinking, smoking and betel quid chewing might have a chance to be randomly misclassified, which usually lead toward the null or underestimation.

Except for the unknown factors that might cause non-random errors, our multivariate models controlled for all the known major risk factors, including viral hepatitis B and C, current alcohol drinking and/or cigarette smoking, and increased BMI were found to be independently associated with abnormal elevation of liver transaminase as shown in Tables 2 and 4. Moreover, all these factors except increased BMI were also found to be associated with liver cirrhosis diagnosed through ultrasonography, which further increased the credibility of this study. Therefore, we concluded that biochemical dysfunction of liver may be independently affected by the above risk factors, including betel chewing. Tables 3 and 4 seemed to indicate that persistent elevation of liver transaminases could be an early sign of chronic inflammation that might result in the liver cirrhosis. But we should not make too strong inference because of the limited number of cases and lack of biopsy proof.

Therefore, we concluded that biochemical dysfunction of liver may be independently affected by the above risk factors, including betel chewing. Efforts to reduce the prevalence of liver disease in Aboriginal should, therefore, include efforts to increase quitting of the betel habit.

The above findings deserve to examine possible causal mechanism between betel quid chewing and liver injury. Areca nut was reported to immunologically suppress body defense to HBV/HCV infection [34] and increase the risk of toxic hepatitis[31,32], which might be related to other reactive oxygen adducts formed as a result of habitual betel chewing [35,36]. An alternative explanation might be the high proportion (up to 37.5%) of betel quids contaminated by aflatoxin [37], which is a well-documented hepatotoxin [38,39]. Further studies are needed to determine which components of betel quids may be responsible for liver damage are now required.

Although Safrole, listed as a carcinogen by the Environmental Protection Administration, USA, have been isolated from areca nut [34], the phenolic compounds in betel leaf might be antimutagenic [40,41] or possibly protective against carcinogens [42]. Thus, it is difficult to hypothesize. As there were 86% smokers and 75% drinkers in Chinese betel chewers [14], the possible mechanisms and interactions among smoking, drinking, and betel chewing deserved further studies on the development of liver cirrhosis.

## Conclusion

The shortened life expectancy in Aboriginal people of Taiwan has long been noticed since the early 1980's [1]. The issue has attracted public concern after democratization of Taiwan. Recently, the government of Taiwan has put a lot of efforts to improve Aboriginal health for the reason of equity and justice. The funding of this study was part of such efforts to determine the major risk factors and prevalence rates of various diseases. The next moves will not only further increase coverage rates of National Health Insurance, improve health manpower and accessibility for healthcare of Aboriginal community in remote area, but also provide programs for reduction in betel chewing rates in Aboriginals to effectively prevent liver diseases

## Competing interests

The authors declare that they have no competing interests.

## Authors' contributions

YC and PH joined in the design of the study. CF and TJ carried out the studies, participated in the sequence alignment and data collection. CF and PH conducted data analysis, and CF drafted the manuscript. JD supervised to complete the data analysis and helped to finalize the manuscript. All of the authors read and approve the manuscript.

## Pre-publication history

The pre-publication history for this paper can be accessed here:


